# Relationships between Clinicopathological Features and Cerebrospinal Fluid Biomarkers in Japanese Patients with Genetic Prion Diseases

**DOI:** 10.1371/journal.pone.0060003

**Published:** 2013-03-28

**Authors:** Maya Higuma, Nobuo Sanjo, Katsuya Satoh, Yusei Shiga, Kenji Sakai, Ichiro Nozaki, Tsuyoshi Hamaguchi, Yosikazu Nakamura, Tetsuyuki Kitamoto, Susumu Shirabe, Shigeo Murayama, Masahito Yamada, Jun Tateishi, Hidehiro Mizusawa

**Affiliations:** 1 Department of Neurology and Neurological Science, Tokyo Medical and Dental University Graduate School of Medical and Dental Sciences, Tokyo, Japan; 2 Department of Molecular Microbiology and Immunology, Nagasaki University Graduate School of Biomedical Sciences, Nagasaki, Japan; 3 Department of Neurology, Aoba Neurosurgical Hospital, Sendai, Japan; 4 Department of Neurology and Neurobiology of Aging, Kanazawa University Graduate School of Medical Science, Kanazawa, Japan; 5 Department of Public Health, Jichi Medical University, Tochigi, Japan; 6 Department of Prion Protein Research, Division of CJD Science and Technology, Tohoku University Graduate School of Medicine, Miyagi, Japan; 7 Center for Health and Community Medicine, Nagasaki University Hospital, Nagasaki, Japan; 8 Department of Neuropathology, Tokyo Metropolitan Institute of Gerontology, Tokyo, Japan; 9 Harukaze Healthcare Service Institution, Fukuoka, Japan; Ohio State University, United States of America

## Abstract

A national system for surveillance of prion diseases (PrDs) was established in Japan in April 1999. Here, we analyzed the relationships among prion protein gene (*PRNP*) mutations and the clinical features, cerebrospinal fluid (CSF) markers, and pathological characteristics of the major genotypes of genetic PrDs (gPrDs). We retrospectively analyzed age at onset and disease duration; the concentrations and incidences of 14-3-3 protein, tau protein, and abnormal prion protein (PrP^Sc^) in the CSF of 309 gPrD patients with P102L, P105L, E200K, V180I, or M232R mutations; and brain pathology in 32 autopsied patients. Three clinical phenotypes were seen: rapidly progressive Creutzfeldt-Jakob disease (CJD), which included 100% of E200K cases, 70% of M232R, and 21% of P102L; slowly progressive CJD, which included 100% of V180I and 30% of M232R; and Gerstmann-Sträussler-Scheinker disease, which included 100% of P105L and 79% of P102L. PrP^Sc^ was detected in the CSF of more than 80% of patients with E200K, M232R, or P102L mutations but in only 39% of patients with V180I. V180I was accompanied by weak PrP immunoreactivity in the brain. Patients negative for PrP^Sc^ in the CSF were older at disease onset than positive patients. Patients with mutations associated with high 14-3-3 protein levels in the CSF typically had synaptic deposition of PrP in the brain and a rapid course of disease. The presence of small PrP protein fragments in brain homogenates was not correlated with other clinicopathological features. Positivity for PrP^Sc^ in the CSF may reflect the pathological process before or at disease onset, or abnormality in the secretion or metabolism of PrP^Sc^. The amount of 14-3-3 protein in the CSF likely indicates the severity of the pathological process and accompanying neuronal damage. These characteristic features of the CSF in cases of gPrD will likely facilitate accurate diagnosis and clinicopathological study of the various disease subtypes.

## Introduction

Genetic prion diseases (gPrDs) are classified into three major phenotypes: fatal familial insomnia (FFI), Gerstmann-Sträussler-Scheinker disease (GSS), and genetic Creutzfeldt-Jakob disease (gCJD). These diseases are characterized by disease-specific mutations in the prion protein gene (*PRNP*), some of which are inherited in an autosomal dominant fashion. More than 30 point mutations and repeated octapeptide insertions have been reported [1]. The frequency of gPrDs and the proportion of each *PRNP* mutation differ from country to country. The E200K mutation is the most common in European countries and the only mutation observed in Slovakia, whereas V210I is the most frequent mutation observed in Italy [2]. In Japan, where a nationwide surveillance system for PrDs was established in April 1999, a 10-year review of PrDs was published in 2010 [3]. Several of the mutations detected, namely V180I, P105L, and M232R, are seen almost exclusively in patients from Japan.

Here, we analyzed in detail the clinical, laboratory, and pathological characteristics of Japanese patients with gPrD. We also investigated biomarkers of prion disease in the cerebrospinal fluid (CSF) of patients with gPrD. Biomarkers including 14-3-3 protein, tau protein, neuron-specific enolase, and S100 protein, are helpful tools for the diagnosis of sporadic CJD [4]. Although these markers previously were investigated in 174 cases of gPrDs, the data were limited to the major mutations found in European countries [4]. In addition, development of the real-time quaking-induced conversion (RT-QUIC) method [5], [6] has enabled us to detect abnormal prion protein (PrP^Sc^) in the CSF. In the current study, we analyzed PrP^Sc^, 14-3-3, and tau proteins in the CSF of patients with gPrD characterized by major mutations found in Japan.

## Methods

### Patients

The CJD Surveillance Committee of Japan diagnosed gPrDs in accordance with the WHO Case Definition Criteria for epidemiological surveillance of these diseases (Table S1). Pathologically confirmed cases were classified as “definite” gPrD and cases diagnosed clinically without pathological analysis were classified as “probable.” From April 1999 through August 2011, 336 patients throughout Japan were categorized as having a gPrD, and we analyzed data from 309 patients with definite or probable gPrD associated with five major mutations: P102L, P105L, V180I, E200K, and M232R. Other mutations, such as D178N-129MM (the causative mutation of FFI) were not included, because few patients had these mutations. Whether M232R actually causes gPrD has not yet been determined definitively [3]; however, we provisionally classified patients with the M232R mutation as having a gPrD, according to the classification convention of the Surveillance Committee of Japan [3].

### Clinical Analyses and Laboratory Examinations

We collected information regarding patients’ age at onset, sex, family history, clinical duration (duration from onset to death, or to the point when we confirmed the condition of the patient if he or she was alive), and clinical signs (dementia, psychological disturbance, cerebellar disturbance, visual disturbance, pyramidal or extrapyramidal signs, myoclonus, and akinetic mutism). Disease onset was defined as the time when the patient started to show one of the aforementioned clinical signs. Patients with the M232R mutation were categorized into two phenotypes: rapid (M232R-rapid) and slow (M232R-slow) [7]. Here, we defined patients with M232R-rapid as those in whom akinetic mutism developed within 7 months of disease onset or those who died less than 8 months after disease onset with or without akinetic mutism. Because divergent clinical and neuropathological phenotypes have been associated with the P102L mutation [8], [9], we categorized patients with P102L into those with the GSS phenotype, who showed cerebellar symptoms first, and those with the CJD phenotype, who showed rapidly progressive dementia.

We evaluated electroencephalograms (EEG) for the appearance of periodic sharp wave complexes (PSWC) and assessed the cerebral cortices, basal ganglia, and thalamus for hyperintensities on diffusion-weighted imaging (DWI), fluid-attenuated inversion recovery (FLAIR) imaging, and T2-weighted magnetic resonance imaging (MRI) [3]. The schedules for the EEG and MRI examinations were determined by each patient’s physician.

Genomic DNA extracted from patients’ blood was used to analyze the open reading frame and polymorphisms of codons 129 and 219 of the *PRNP* gene [10]. We classified the family history as “definite” when any member of the patient’s family had the same mutation as the patient and as “possible” when any member of the patient’s family had a prion disease with an unknown mutation or dementia due to neurodegenerative disease (see Table S2).

### CSF Examinations

Analysis was performed on available CSF samples stored at Nagasaki University [3], [5], [10], [11], obtained from patients with the P102L, P105L, V180I, E200K, or M232R mutations. Although an assay for 14-3-3 protein in the CSF has been described previously, the method was not standardized until April 2009 [3]. Here we reevaluated 14-3-3 protein levels in the CSF with Western blotting by using polyclonal antibodies specific for the γ isoform of 14-3-3 (dilution, 1∶500; catalog no. 18647, IBL, Gunma, Japan) and with semiquantitative analysis by using chemiluminescence (model no. LAS-3000, FufiFilm, Tokyo, Japan) [11]. Total tau protein was measured by means of an ELISA, as previously described [12]. PrP^Sc^ in the CSF was detected by using RT-QUIC, as previously described [5]; negative results were confirmed by performing a second QUIC reaction. For RT-QUIC analysis of the CSF of patients with gPrDs, we also used a recombinant prion protein with or without the aforementioned mutations. The time between disease onset and acquisition of the CSF sample was noted. We used CSF samples from patients with definite MM1-type sporadic CJD as positive controls and those from healthy adults as negative controls.

### Neuropathological Examinations and Western Blot Analysis of PrP^Sc^

Of the 184 patients who had died, 32 were autopsied and their brains examined histopathologically. Brain tissues obtained from the autopsies were sectioned by using routine neuropathological techniques and stained with hematoxylin and eosin. Immunohistochemistry for PrP^Sc^ was performed by using mouse monoclonal antibodies specific for the PrP^Sc^ protein (3F4) [13]. Frozen samples of brain tissue were homogenized and evaluated in Western blot analyses by using the 3F4 antibody to determine PrP^Sc^ levels [14].

### Statistical Analysis

The Mann–Whitney *U* test was used to compare patient groups in regard to age at onset, disease duration, and level of tau protein in the CSF. Fisher’s exact probability test was used for comparisons of sex, rate of occurrence of each clinical sign, presence of PSWC on EEG, presence of hyperintensity on MRI sequences, and rates of positive detection of 14-3-3 and PrP^Sc^ proteins. For analysis of the correlation between CSF markers and each clinical parameter, Student’s *t*-test or analysis of variance (ANOVA) was used. Significance was defined as *P*<0.05. All analyses were performed by using Prism 5 software (GraphPad Software, La Jolla, CA) and IBM SPSS Statistics (IBM, New York, NY).

### Ethical Issues

The family of each patient gave informed consent for patient inclusion in the study. The study protocol was approved by the Institutional Ethics Committees of Kanazawa University and Tokyo Medical and Dental University.

## Results

### Clinical Features

The clinical characteristics of all patients are summarized in Table S2, categorized by *PRNP* mutation. All of the patients with the P105L mutation exhibited clinical features of GSS. Of the 57 patients with P102L, 45 manifested the GSS phenotype (P102L-GSS), and 12 had the slowly progressive CJD phenotype (P102L-CJD). All patients with E200K and 33 patients with M232R manifested the rapidly progressive CJD phenotype. The slowly progressive CJD phenotype was seen in 14 patients with M232R (M232R-slow) and all patients with V180I.

**Figure 1:**
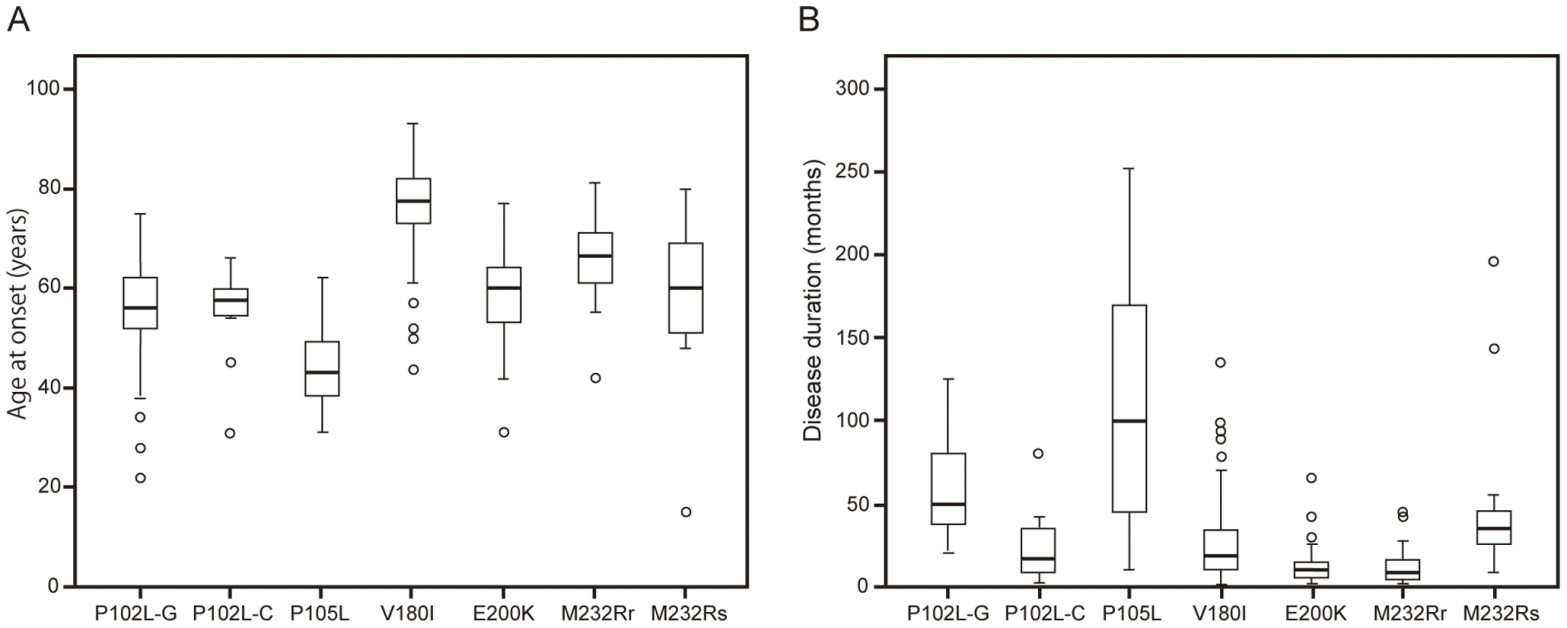
Age of onset and disease duration. Box-and-whisker plots show (A) age at onset and (B) disease duration for each type of gPrD (P102L-GSS, P102L-CJD, P105L, V180I, E200K, M232R-rapid, and M232R-slow). The horizontal line inside each box indicates the median value, and the length of the box is the interquartile range (from 25th to 75th percentile). The extremes of the whiskers contain 95% of values. Open circles indicate outliers. P102L-G, P102L GSS type; P102L-C, P102L CJD type; M232Rr, M232R-rapid; M232Rs, M232R-slow.

There were no significant differences in the male-to-female ratios among the mutations (*P*>0.05; Fisher’s exact probability test). The range in median age at onset was wider for our patients than was that of gPrD patients in European countries [2]; our youngest group of patients was 44.3 years at an average disease onset (patients with P105L), and our oldest group of patients (who had V180I) was 76.5 years at an average (Figure 1A and Table S2). Relative to patients with the CJD phenotype (gCJD), those with the GSS phenotype (P102L-GSS, P105L) had significantly longer disease duration and less frequent myoclonus (Figure 1B and Table S2; *P*<0.005; Mann–Whitney *U* test). Among patients with gCJD, those with E200K or M232R-rapid showed clinical courses typical of classical CJD, but patients with P102L-CJD, V180I, or M232R-slow showed atypical clinical features, including prolonged duration (Figure 1B and Table S2). Myoclonus was less frequent in patients with V180I or M232R-slow than in those with E200K or M232R-rapid (*P*<0.005; Fisher’s exact probability test). In addition, patients with the V180I mutation had a lower incidence of cerebellar signs than did those with the E200K or M232R-rapid mutation (*P*<0.005; Fisher’s exact probability test).

Relative to patients with any other mutation, patients with E200K or M232R-rapid showed a higher incidence of PSWCs on EEG (*P*<0.005; Fisher’s exact probability test). Hyperintensity on MRI was frequently observed in gCJD patients, especially those with V180I, but was rare in GSS patients (P102L-GSS, P105L vs. all other mutations; *P*<0.05; Fisher’s exact probability test, Table S2).

Unlike European patients [15], most (92%) normal Japanese adults are methionine homozygous at codon 129 (129 MM), with only 8% who are methionine-valine heterozygous (129 MV). Patients with the P102L or M232R mutation showed the same distribution of codon 129 polymorphisms as that of the normal population. All patients with the E200K mutation had the 129 MM variant, and all patients with the P105L mutation had 129 MV heterozygosity, with the 129 V codon on the same allele as the 105 L codon. Although 24% of patients with V180I had 129 MV heterozygosity, clinical features did not differ between patients with the 129 MM and 129 MV variants (data not shown). Four patients (1 with P102L, 2 with E200K, and 1 with M232R) had 219 EK heterozygosity, and the rest had 219 EE homozygosity.

Patients with the P102L, P105L, or E200K mutation showed a high frequency of familial PrDs, although none of the patients with M232R and only 1 patient with V180I had a “definite” family history of PrD (Table S2).

### Clinical Features and Biomarkers in CSF

**Figure 2:**
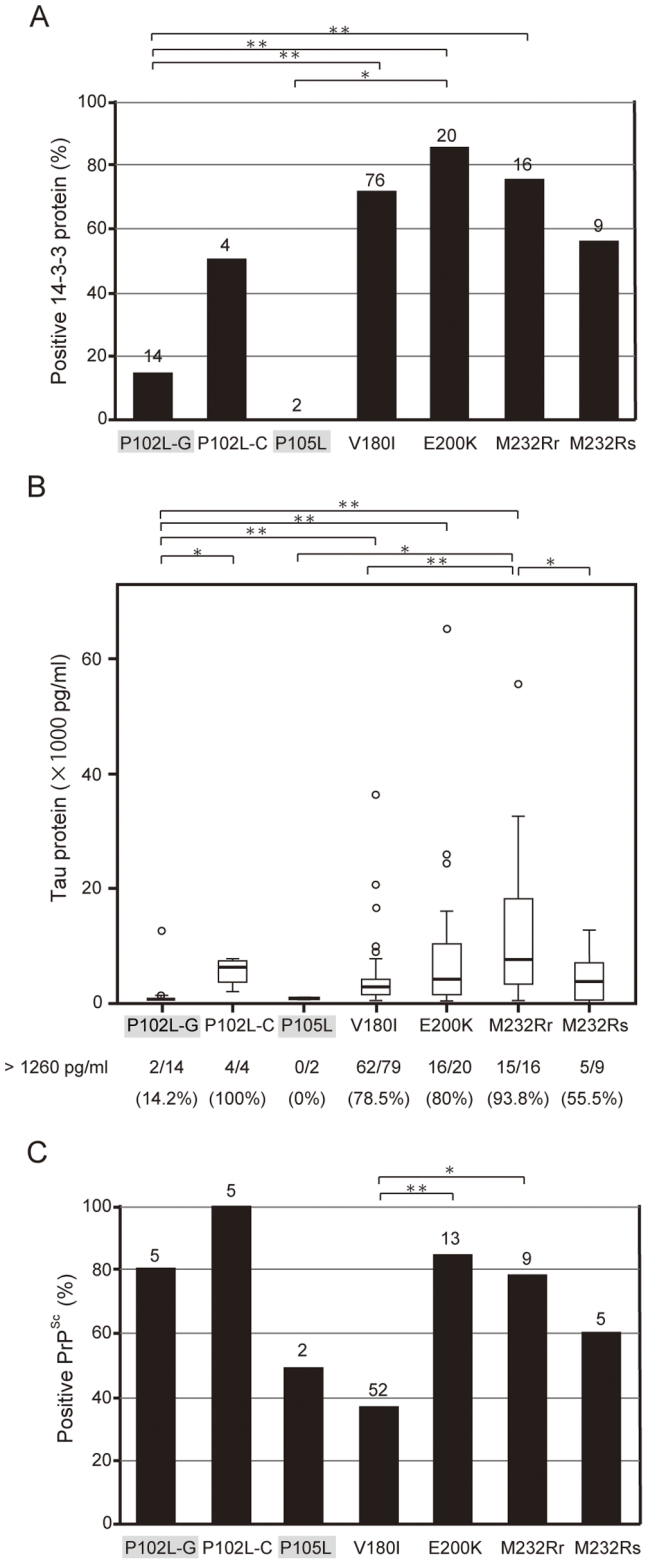
Relationships between CSF biomarkers and genotypes. (A) Rates of positivity of 14-3-3 protein in the CSF of 141 gPrD patients. The number of patients for whom data were available is shown at the top of each column. (B) Box-and-whisker plots of the concentrations of tau proteins in the CSF of 144 gPrD patients. The number of patients with a tau concentration of 1260 pg/mL or greater and the detection rate for each mutation are shown below the graph. (C) Positivity rates of PrP^Sc^ protein in the CSF of 91 gPrD patients. *P<0.05; **P<0.005 (Fisher’s exact probability test).

CSF was positive for the 14-3-3 protein in 85% of patients with E200K and in 50% to 75% of the remaining patients with gCJD; however, this protein was rarely present in the CSF of patients with GSS (P102L-GSS, 14.2%; P105L, 0%; Figure 2A). The concentration of tau protein in CSF was highest (14,215±15,058 pg/mL) in patients with M232R-rapid and lowest (711±214 pg/mL) in patients with P105L (Figure 2B). The rate of tau protein positivity (that is, exceeding the cut-off level of 1260 pg/mL [12]) was 78.5% or greater in patients with P102L-CJD, V180I, E200K, or M232R-rapid but less than 15% in patients with GSS (shaded in Figure 2B). PrP^Sc^ was detected in the CSF of most patients with P102L (-GSS; 80% and -CJD; 100%), E200K (84.6%), or M232R-rapid (77.8%; Figure 2C); these rates are comparable to those of patients with sporadic CJD (sCJD) [5]. The rate of PrP^Sc^ positivity was much lower in patients with V180I (39%) than in patients with sCJD [5], [16], E200K, or M232R-rapid (*P*<0.05; Fisher’s exact probability test).

**Figure 3:**
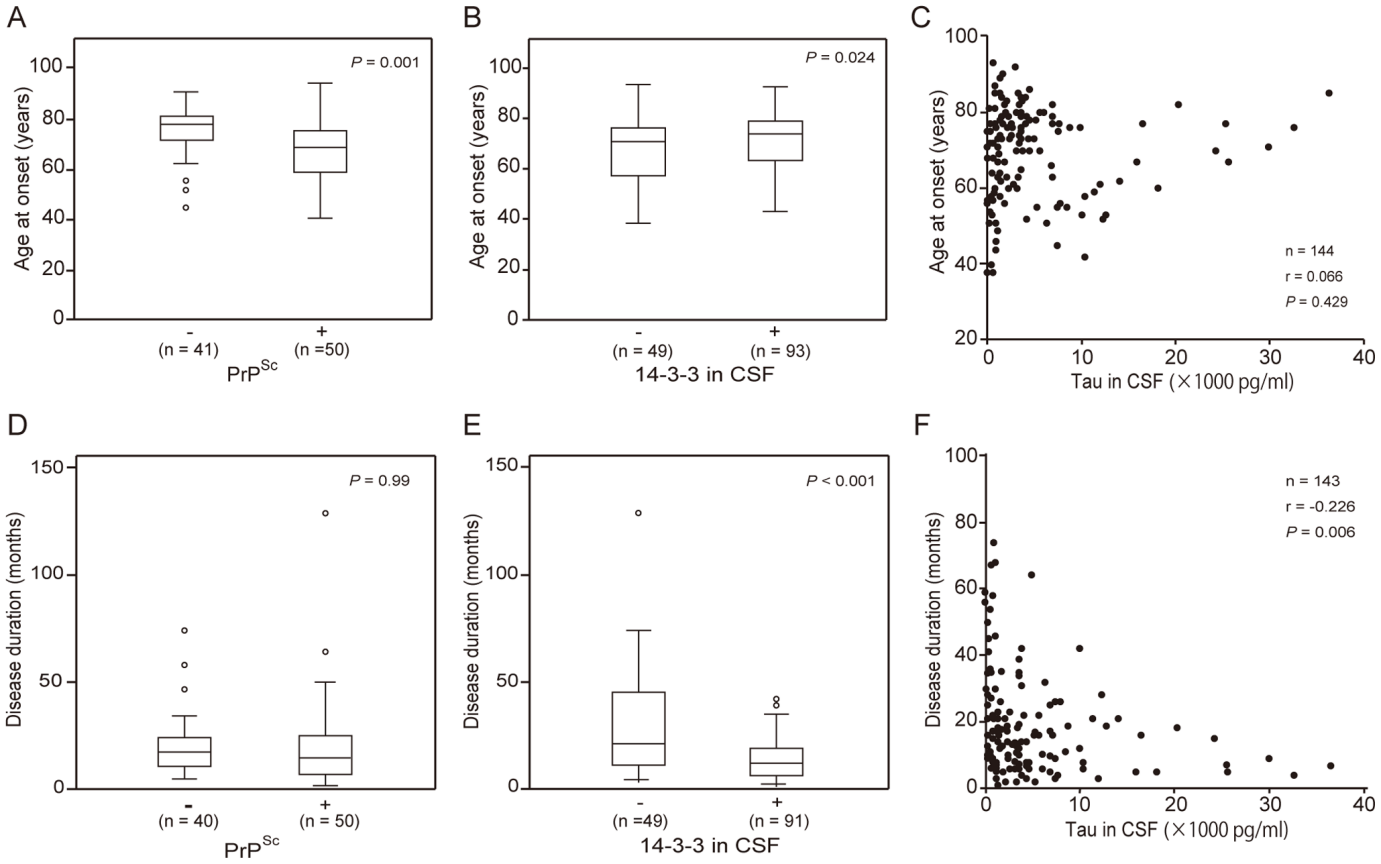
Relationships between CSF biomarkers and clinical features. (A–C) Age at onset and (D–F) disease duration (time from disease onset to death or to when the condition was confirmed) were compared between patients with and without (A, D) PrP^Sc^ protein or (B, E) 14-3-3 protein. In addition, (C, F) the correlation between these clinical features and the concentration of tau protein in CSF was examined for patients with the P102L, P105L, V180I, E200K, or M232R mutation.

To investigate associations between CSF biomarkers and clinical features, we analyzed correlations between age at onset, disease duration, and each CSF biomarker. We also investigated whether the positivity of CSF biomarkers varied with the time that the sample was acquired relative to disease onset. The date of CSF analysis was obtained from the records of 124 gPrD patients (P102L-GSS, 11 cases; P102L-CJD, 4 cases; P105L, 2 cases; V180I, 71 cases; E200K, 16 cases; M232R-rapid, 13 cases; and M232R-slow, 7 cases). Age at onset was significantly greater in PrP^Sc^-negative patients than in PrP^Sc^-positive patients (Figure 3A; *P* = 0.001) and in 14-3-3-positive patients than in 14-3-3-negative patients (Figure 3B; *P*<0.05) but was not associated with the concentration of tau protein (Figure 3C). Disease duration was not correlated with PrP^Sc^ positivity but was shorter in 14-3-3-positive patients than 14-3-3-negative patients (Figure 3E; *P*<0.001) and patients with high tau concentration (Figure 3F; *r* = –0.226). The interval from disease onset to the date of CSF examination was shorter in 14-3-3-positive patients than in 14-3-3-negative patients (Figure S1B; *P*<0.001) and was not correlated with the concentration of tau protein or PrP^Sc^ positivity in CSF samples (Figure S1A, C). The positive correlation between the interval from disease onset to the date of CSF examination and disease duration was significant (*r* = 0.835; data not shown).

**Figure 4:**
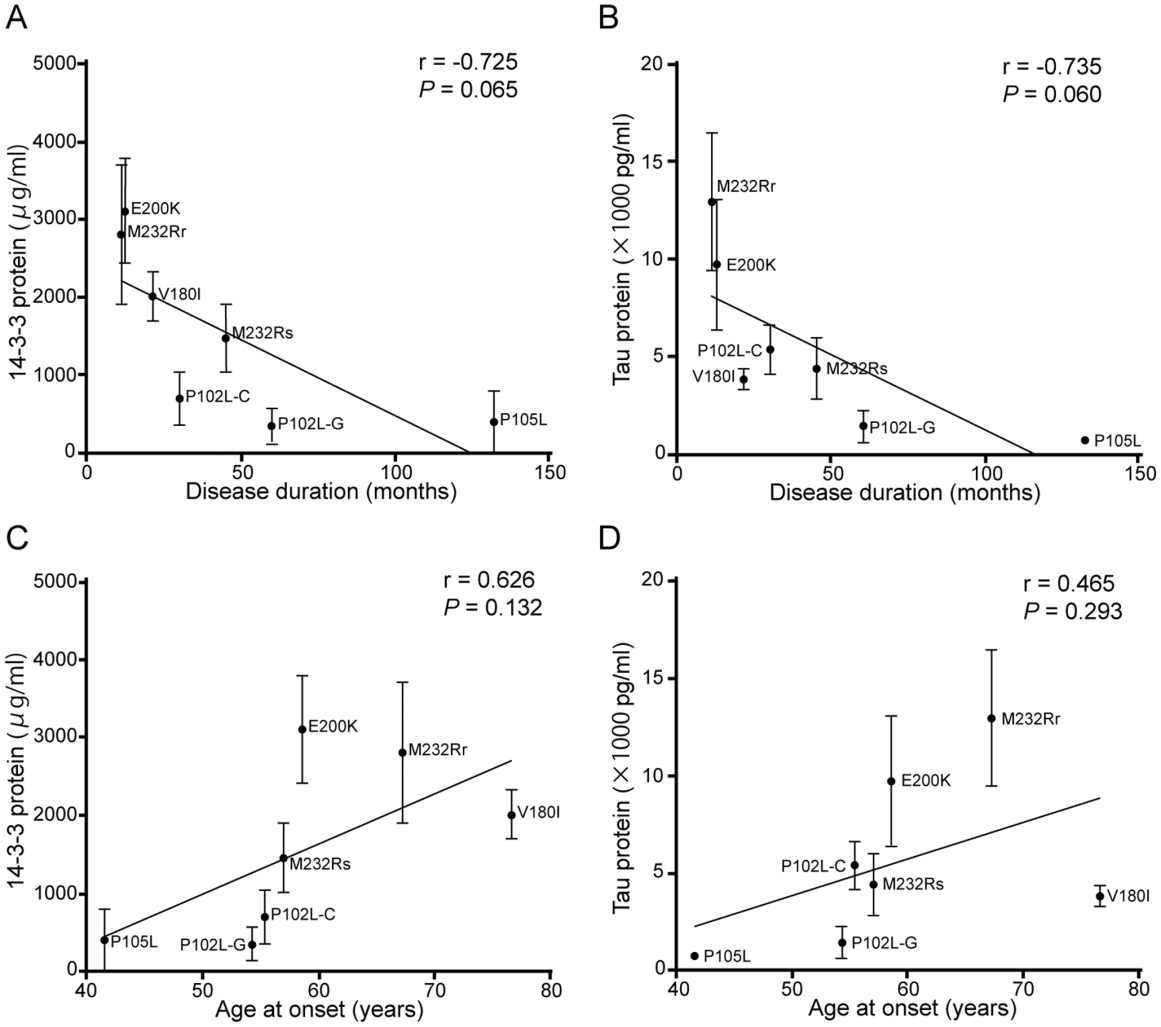
Linear regression analysis of *PRNP* mutations. The correlation between average concentration of 14-3-3 (A and C) or tau (B and D) protein in the CSF with disease duration (mean ± standard error; A and B) or age at onset (C and D) for each mutation was analyzed by using linear regression. P102L-G, P102L GSS type; P102L-C, P102L CJD-type; M232Rr, M232R-rapid; M232Rs, M232R-slow.

Disease duration decreased with increasing 14-3-3 or tau protein concentration, depending on the mutation present (Figure 4A, B), and there was a trend toward a positive correlation between these biomarkers and age at onset, as analyzed by mutation (Figure 4C, D).

### Western Blot Analysis of Brain Homogenates and Biomarkers in CSF

**Figure 5:**
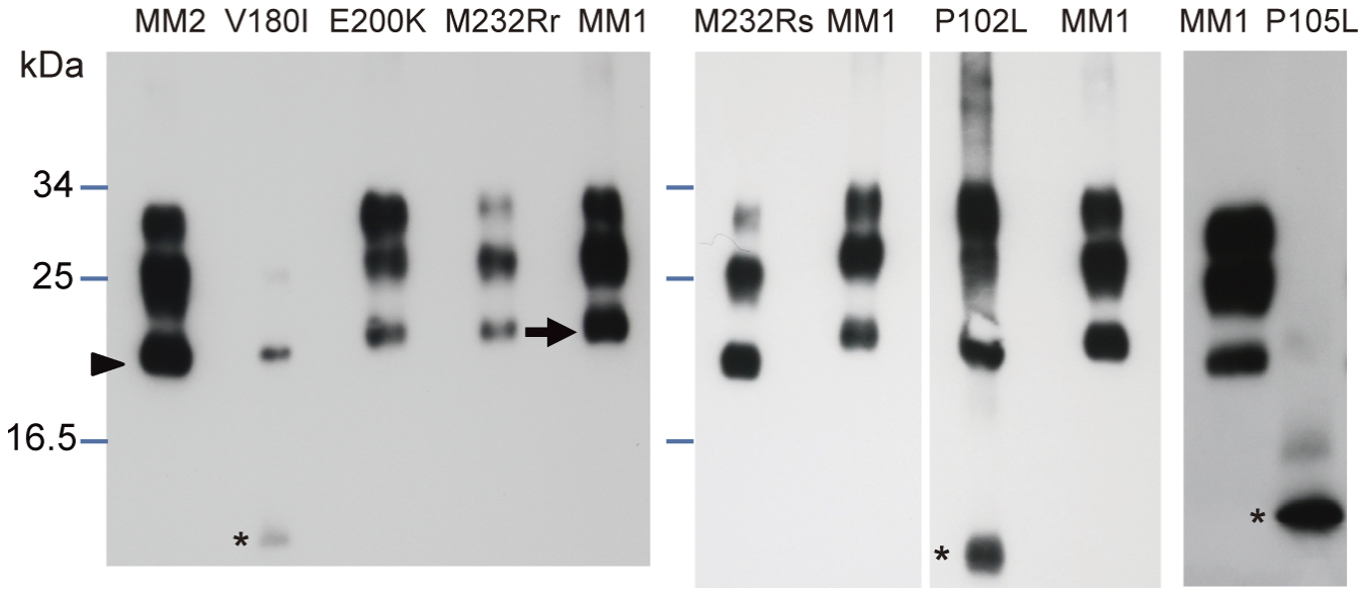
Western blot analysis of PrP^Sc^ in PK-treated brain homogenates. MM2-type sCJD, V180I, E200K (1∶30 dilution of homogenate), M232R-rapid (1∶10 dilution), M232R-slow, P102L-GSS, and P105L (a different case from that in Figure 2). The 3 different molecular-weight bands from large to small correspond to di-, mono-, and unglycosylated fragments. Bands with a shorter migration distance indicate PrP^Sc^ subtype 1 (arrow), which is processed at residues 74–97, whereas bands with a longer migration distance indicate subtype 2 (arrowhead), which is processed at residues 82–102. There were additional bands of smaller molecular weight in the V180I, P102L-GSS, and P105L samples (asterisks).

Western blot analyses of proteinase K (PK)-treated PrP^Sc^ typically show three bands that correspond to the di-, mono-, and unglycosylated PrP^Sc^ fragments [17]. The two subtypes of PrP^Sc^ differ in the molecular weight of the unglycosylated PrP^Sc^: subtype 1 (21 kDa) is cleaved by PK primarily at residue 82 (the range of residues 74–97 is possible PK digestion sites) of PrP^Sc^ (Figure 5, MM1 type), whereas subtype 2 (19 kDa) is cleaved primarily at residue 97 (residues 82–102; Figure 5, MM2 type). Subtype 1 PrP^Sc^ bands were detected in brain homogenates from our patients with E200K, M232R-rapid, or P102L-GSS (Figure 5). Subtype 2A PrP^Sc^ bands were detected in brain homogenates from patients with V180I or M232R-slow, although V180I cases showed only weak bands of the mono- and unglycosylated fragments (Figure 5), as reported previously [18]. An additional low-molecular-weight (<7 kDa) band was detected in brain homogenates from patients with V180I or P102L-GSS. Whereas a faint band that corresponds to a <6-kDa PK-resistant fragment has been reported previously to occur in cases with P105L [19], we detected a prominent band at ∼7 kDa in brain homogenates from our patients with P105L.

Bands associated with the typical PK-resistant PrP^Sc^ fragments were weak or undetectable in patients with P105L or V180I; these patients also were characterized by low rates of PrP^Sc^ positivity in the CSF. We did not find any relationship between the band pattern on Western blots and 14-3-3 positivity or tau protein concentration in CSF.

To exclude the possibility that the RT-QUIC procedure is not well-suited for amplifying the PrP^Sc^ conformer associated with the V180I mutation, we performed RT-QUIC by using a recombinant prion protein with or without the V180I mutation and CSF from patients with V180I CJD. The PrP^Sc^ positivity rate in the CSF of these patients, measured with the RT-QUIC method by using recombinant prion protein, was the same with or without the mutation (data not shown).

### Neuropathology, Immunohistochemistry, and Biomarkers in CSF

Only 32 (17.3%) of the 184 patients who died were autopsied; several of these cases have been described previously [7], [20]–[22]. Usually, the cerebral cortices of patients with the V180I mutation showed typical spongiform changes, neuronal loss, and astrocytosis (Figure S2A); destructive changes (status spongiosus) were rarely observed, however, even in cases of relatively short clinical duration. Immunohistochemistry of patients with V180I showed fairly weak synaptic-type PrP deposition (Figure S2B) [20]. The brain tissues of patients with E200K (Figure S2C, D) or M232R-rapid (Figures S2E, F) showed typical spongiform changes and synaptic-type PrP deposition, resembling those of patients with MM1-type sCJD [6], [19]. Neuropathological findings in patients with M232R-slow showed large, confluent, vacuole-type spongiform changes and perivacuolar-type PrP deposition in the cortex (Figures S2G, H). These features differed from those in MM1-type sCJD and resembled those in MM2 cortical-type sCJD [6], [18]. In patients with P105L, we found multiple PrP-positive amyloid plaques and diffuse PrP deposition in the deep layers of the cerebral cortex without spongiform changes, consistent with a previous report [19]. Cases of P102L have been associated with PrP-positive amyloid plaques in the cerebral cortex with or without spongiform changes or synaptic-type deposits [23], [24].

The low PrP^Sc^ positivity in the brains of patients with the V180I mutation was consistent with the weak immunoreactivity observed in Western blotting and immunohistochemistry (Figure 5 and Figure S2B). The cerebral cortices of patients with the V180I, E200K, or M232R mutation showed typical spongiform changes (Figure S2), which also were associated with a high concentration of 14-3-3 or tau protein in CSF. Patients with synaptic-type of PrP deposition in the cerebral cortex (V180I, E200K, or M232R-rapid) were older at disease onset (53.7±13.6 years vs. 71.1±10.9 years; *P*<0.001) and had shorter disease duration (59.4±44.6 months vs. 17.9±15.9 months; *P*<0.001) than did patients with plaque-type PrP deposition (P102L, P105L, or M232R-slow).

## Discussion

The mutations and clinical characteristics associated with gPrDs in Japan differ from those in European countries [2], [3]. Although clinical variation is observed, more than 60% of the gPrD cases we examined had mutations of V180I, P105L, and M232R; these mutations were mostly reported from Japan [3]. Patients with P105L were characterized by onset at a young age, prolonged clinical duration, spastic paraparesis, and dementia; however, clinical variation is sometimes observed [25], [26]. PSWC on EEG and hyperintensity on MRI studies are usually negative in patients with P105L. Relative to patients with E200K, those with P102L-CJD or V180I showed slower disease progression, with less myoclonus and fewer PSWC on EEG (Table S2; *P* = 0.033 and *P*<0.0001, respectively). Among all patients with gPrDs, those with V180I typically are the oldest at disease onset, have the fewest cerebellar symptoms, and show a characteristic pattern of lesions on MRI [27]. Although a few patients with V180I [28] or M232R [29], [30] mutations have been reported from Korea [26], [27] and China [28], long-term nationwide surveillance in Asia has been attempted only in Japan and Taiwan; therefore, further accumulation of cases is needed before ethnic variability can be discussed. Controversy regarding whether the M232R mutation actually causes gPrD remains, not only because patients with this mutation have no family history of the disease, but also because this amino acid substitution has been discovered in a few patients with dementia with Lewy bodies as well as several healthy individuals [31]. Here, we provisionally classified the disease associated with the M232R mutation as gPrD and analyzed its clinical and pathological features.

Few reports have correlated clinical or pathological features of gPrDs and CSF biomarkers of the disease [4], [32]. Here, we investigated the presence of 14-3-3, tau, and PrP^Sc^ proteins in the CSF of patients with gPrD. To our knowledge, the patient population in the current study is the largest in which CSF levels of PrP^Sc^ have been examined in gPrDs. The low number of patients with V180I for whom PrP^Sc^ in the CSF was positive suggests that either little PrP^Sc^ is produced with this mutation or that the mutant protein is unstable; each possibility is consistent with the weak immunoreactivity seen in the Western blots of brain homogenates and immunohistochemistry. However, we need to perform RT-QUIC by using various concentrations of brain homogenate from patients with V180I to confirm the relationship between the quantity of PrP^Sc^ and the sensitivity of RT-QUIC for detecting PrP^Sc^ in the CSF. In this regard, we already have used recombinant prion proteins with or without the target mutation to confirm that brain PrP^Sc^ from patients with P102L, V180I, or M232R is amplified efficiently by RT-QUIC.

Like those with V180I, few of our patients with P105L were positive for PrP^Sc^ in their CSF (Figure 2C). Nonetheless, we will need to analyze more patients with this mutation to confirm this finding. Given that the Western blots of brain homogenates from patients with P105L showed only low-molecular-weight PK-resistant bands, the physiology of PrP^Sc^ in patients with the P105L mutation likely differs from that in patients with other mutations, in terms of the behavior of the mutant protein as a prion [33].

Disease duration was shorter in 14-3-3-positive patients than in 14-3-3-negative ones, regardless of whether the data were analyzed by patient (Figure 3E) or by mutation (Figure 4A). In addition, tau protein concentration was negatively correlated with disease duration (Figures 3F, 4B). The presence of 14-3-3 and tau protein likely reflects the severity or speed of progression of the disease; in pathological analysis, increased 14-3-3 protein levels in patients and increased tau concentrations were seen with mutations associated with synaptic-type PrP deposition, such as V180I, E200K, and M232R-rapid (Figure S2B, D, F). Our results are consistent with those of a previous analysis of patients with the E200K mutation and sCJD in Israel [32]; both indicate that these markers leak into the CSF in the presence of acute brain damage.

To examine the relationship between biomarker positivity and disease stage, we measured the interval from disease onset to acquisition of the CSF sample (Figure S1). The presence of 14-3-3 or tau is reportedly unrelated to the timing of CSF sampling during the clinical course of disease progression (that is, early, middle, and advanced stages) [4]. However, in the current study, CSF samples were obtained during an earlier stage in 14-3-3-positive patients compared with 14-3-3-negative patients. This difference likely reflects that 14-3-3-positive patients experienced more rapid disease progression and came to the hospital sooner than did 14-3-3-negative patients; this explanation is supported by our finding that disease duration was strongly correlated with the length of the interval from disease onset to CSF examination (*r* = 0.835). We were able to obtain data on disease duration and the timing of CSF sampling but not on disease stage at the time of sampling; therefore, whether measuring CSF levels of 14-3-3 protein during the early phase of gPrDs is useful is difficult to determine.

In the current study population, age at disease onset was greater in 14-3-3-positive patients than in 14-3-3-negative ones (Figure 3B), consistent with the data in one previous study [32] but not another [4]. However, whereas age at onset was lower in PrP^Sc^-positive patients than in PrP^Sc^-negative patients, disease duration was not correlated with PrP^Sc^ positivity (Figure 3D). This result suggests that the detection of PrP^Sc^ does not reflect rapid disease progression after the onset of clinical disease. Although PrP^Sc^ is a marker of a pathological process evaluated after disease onset, perhaps a low PrP^Sc^ level in the CSF is associated with slow disease progression during the preclinical stage. Alternatively, the amount of PrP^Sc^ in the CSF may reflect abnormalities in the secretion or metabolism of the mutant proteins. This hypothesis is consistent with the very weak immunoreactivity for PrP^Sc^ in the brains of patients with V180I, which was associated with the oldest age at onset among all mutations. In a clinicopathological study of P102L patients, destructive (e.g., spongiform) changes in the brain were not always consistent with the degree of PrP^Sc^ deposition [9], [23], indicating that cofactors other than the amount or aggregation of PrP^Sc^ may play an important role in the process of disease onset and progression. Other possibilities are that the central nervous system excretes abnormal PrP into the CSF (as a self-protective mechanism) or produces protective factors more strongly in younger patients. Further investigation is needed to clarify whether additional factors that promote pathological changes or that protect the brain affect disease onset and progression in patients with gPrD.

In conclusion, the presence or amount of CSF biomarkers in patients with genetic prion disease varies with the *PRNP* mutation. Levels of the 14-3-3 and tau proteins are likely to reflect the severity of brain damage, and measurement of these markers may therefore be useful in the diagnosis of gCJD. The absence of PrP^Sc^ in the CSF may be related to older age at onset. RT-QUIC for the detection of PrP^Sc^ in the CSF is a helpful tool for diagnosing gPrDs as well as sCJD, but we note that its sensitivity regarding the V180I mutation is not yet determined. These CSF data, together with characteristic differences in clinical and pathological phenotypes, will help in determining the pathologic mechanisms of these intractable neurodegenerative diseases.

## Supporting Information

Figure S1 **Relationship between CSF biomarkers and the interval from disease onset to the date of CSF examination.** The interval between disease onset and date of CSF examination was compared between patients with or without (A) PrP^Sc^ or (B) 14-3-3 protein. (C) The correlation between this interval and tau protein concentration was examined.
(PDF)

Figure S2 **Hematoxylin and eosin staining, and immunohistochemistry with anti-PrP antibody in the cerebral cortex.** (A, C, E, G) Hematoxylin and eosin staining, as well as (B, D, F, H) immunohistochemistry with an anti-PrP antibody of brain sections from the temporal lobe of patients with *PRNP* mutations. (A) Samples from patients with the V180I mutation showed typical spongiform changes, mild neuronal loss, and astrocytosis. (B) PrP immunostaining of samples from patient with V180I showed very weak synaptic-type PrP immunoreactivity. Samples from patients with the E200K mutation showed (C) typical spongiform changes and (D) synaptic-type PrP deposition. (E) Samples from patients with the M232R-rapid (M232R-R) mutation also showed spongiform changes, as well as neuronal loss and proliferation of hypertrophic astrocytes in the cortex. (F) Immunohistochemistry of patients with M232R-R showed synaptic-type PrP accumulation in the cortex. Pathological changes in samples from patients with the M232R-slow (M232R-S) mutation were different from those of other mutations and included (G) large, confluent, vacuole-type spongiform changes and (H) perivacuolar-type PrP deposits. Scale bar, 300 µm.
(PDF)

Table S1 **WHO Case Definition Criteria for epidemiological surveillance of gPrDs**
(DOC)

Table S2 **Clinical characteristics of patients with gPrD.**
(DOC)
