# Supplementary figures and images for: Relationships between Clinicopathological Features and Cerebrospinal Fluid Biomarkers in Japanese Patients with Genetic Prion Diseases
Source: PLoS One. 2013 Mar 28;8(3):e60003. doi: 10.1371/journal.pone.0060003 (PMC3610658; doi:10.1371/journal.pone.0060003)

A

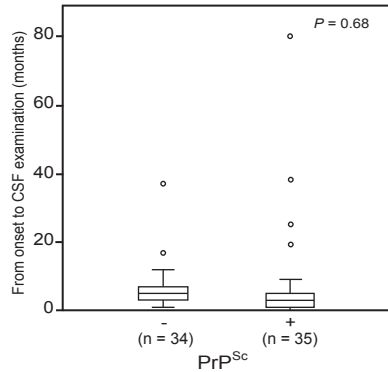

B

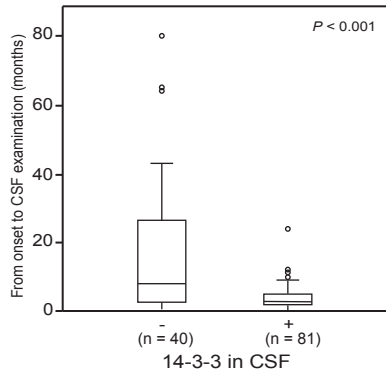

C

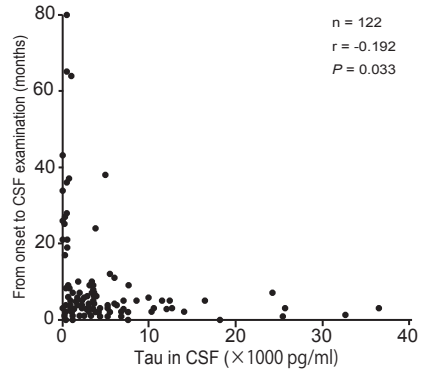

Supplement: Figure S1 — Relationship between CSF biomarkers and the interval from disease onset to the date of CSF examination. The interval between disease onset and date of CSF examination was compared between patients with or without (A) PrPSc or (B) 14-3-3 protein. (C) The correlation between this interval and tau protein concentration was examined. (PDF) [file pone.0060003.s001.pdf]

HE

PrP

V180I

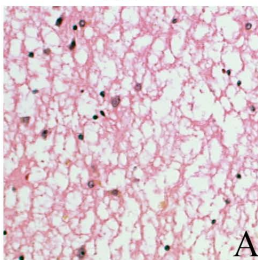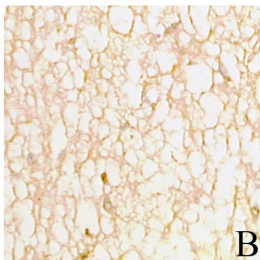

E200K

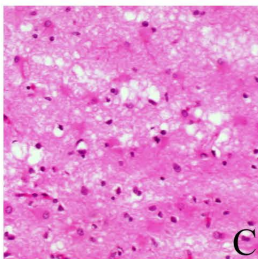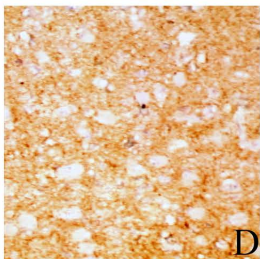

M232R-R

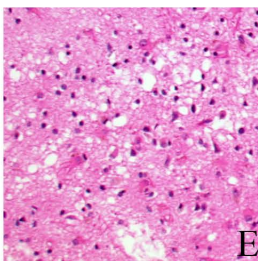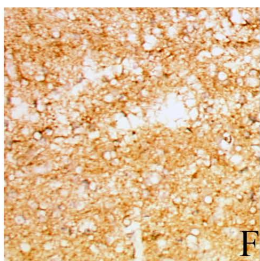

M232R-S

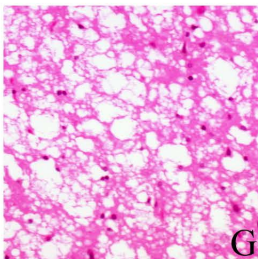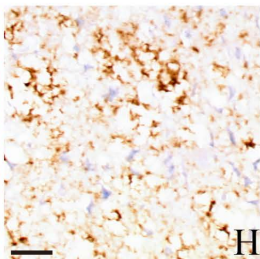

Supplement: Figure S2 — Hematoxylin and eosin staining, and immunohistochemistry with anti-PrP antibody in the cerebral cortex. (A, C, E, G) Hematoxylin and eosin staining, as well as (B, D, F, H) immunohistochemistry with an anti-PrP antibody of brain sections from the temporal lobe of patients with PRNP mutations. (A) Samples from patients with the V180I mutation showed typical spongiform changes, mild neuronal loss, and astrocytosis. (B) PrP immunostaining of samples from patient with V180I showed very weak synaptic-type PrP immunoreactivity. Samples from patients with the E200K mutation showed (C) typical spongiform changes and (D) synaptic-type PrP deposition. (E) Samples from patients with the M232R-rapid (M232R-R) mutation also showed spongiform changes, as well as neuronal loss and proliferation of hypertrophic astrocytes in the cortex. (F) Immunohistochemistry of patients with M232R-R showed synaptic-type PrP accumulation in the cortex. Pathological changes in samples from patients with the M232R-slow (M232R-S) mutation were different from those of other mutations and included (G) large, confluent, vacuole-type spongiform changes and (H) perivacuolar-type PrP deposits. Scale bar, 300 µm. (PDF) [file pone.0060003.s002.pdf]
