# Supplementary material for: Relationships between Clinicopathological Features and Cerebrospinal Fluid Biomarkers in Japanese Patients with Genetic Prion Diseases
Source: PLoS One. 2013 Mar 28;8(3):e60003. doi: 10.1371/journal.pone.0060003 (PMC3610658; doi:10.1371/journal.pone.0060003)
Supplement: Table S1 — WHO Case Definition Criteria for epidemiological surveillance of gPrDs (DOC) [file pone.0060003.s003.doc]

**Table S1. WHO Case Definition Criteria for epidemiological surveillance of gPrDs**

| 1. Sporadic CJD  (a) Possible CJD:  · Progressive dementia; and  · EEG atypical or not known; and  · Duration <2 years; and  · At least 2 out of the following 4 clinical features: myoclonus, visual, or cerebellar disturbance; pyramidal/extrapyramidal dysfunction; akinetic mutism  (b) Probable CJD: (in the absence of an alternative diagnosis from routine investigation)  · Progressive dementia; and  · At least 2 of the following 4 clinical features: myoclonus, visual, or cerebellar disturbance; pyramidal/extrapyramidal dysfunction; akinetic mutism; and  · A typical EEG, whatever the clinical duration of the disease; and/or  · A positive 14-3-3 assay for CSF and a clinical duration to death <2 years  (c) Confirmed (definite) CJD:  · Neuropathological confirmation; and/or  · Confirmation of protease-resistant prion protein (PrP) (immunocytochemistry or Western blot); and/or  · Presence of scrapie-associated fibrils  2. Familial CJD  · Confirmed or probable CJD plus confirmed or probable CJD in a first-degree relative; and/or  · Neuropsychiatric disorder plus disease-specific PrP mutation  Note: For purposes of surveillance, includes Gerstmann-Sträussler-Scheinker (GSS) syndrome and fatal familial insomnia (FFI). |
| --- |
