# Supplementary material for: Relationships between Clinicopathological Features and Cerebrospinal Fluid Biomarkers in Japanese Patients with Genetic Prion Diseases
Source: PLoS One. 2013 Mar 28;8(3):e60003. doi: 10.1371/journal.pone.0060003 (PMC3610658; doi:10.1371/journal.pone.0060003)
Supplement: Table S2 — Clinical characteristics of patients with gPrD. (DOC) [file pone.0060003.s004.doc]

**Table S2. Clinical characteristics of patients with gPrD.**

|  | Mutation | | | | | | | |
| --- | --- | --- | --- | --- | --- | --- | --- | --- |
|  | P102L-GSS | P102L-CJD | P105L | | V180I | E200K | M232R-rapid | M232R-slow |
| No. of patients (% of total) | 45 (13.4%) | 12 (3.6%) | 8 (2.4%) | | 151 (44.9%) | 46 (13.7%) | 33 (9.8%) | 14 (4.2%) |
| No. of men/no. of women | 20/25 | 7/5 | 6/2 | | 58/93 | 26/30 | 15/18 | 8/6 |
| Age at onset (mean ± SE; years) | 55.1±11.5 | 55.4±9.4 | 44.3±9.5 | | 76.5±7.7 | 58.6±9.1 | 66.2±7.7 | 57.6±15.4 |
| Duration of disease (mean ± SE; months) | 58±28.8 | 27±22.2 | 111.2±82.4 | | 25±21.3 | 12.8±11.2 | 12.5±10.9 | 49.9±53.8 |
| Clinical symptoms (No. with symptom/total no. evaluated; % positive among total evaluated) | | | |  | |  |  |  |
| Myoclonus (%) | 9/45 (20.0) | 6/11 (54.5) | 3/7 (42.9) | 57/128 (44.5) | | 37/45 (82.2) | 30/31 (96.8) | 7/14 (50) |
| Akinetic mutism (%) | 16/44 (36.4) | 10/11 (91.0) | 5/7 (71.4) | 66/134 (49.3) | | 40/44 (91.0) | 29/31 (93.5) | 7/14 (50) |
| Dementia (%) | 26/45 (57.8) | 12/12 (100) | 6/6 (100) | 136/137 (99.3) | | 45/45 (100) | 32/32 (100) | 12/14 (85.7) |
| Psychiatric disorder (%) | 17/43 (39.5) | 8/11 (72.7) | 4/7 (57.1) | 67/125 (53.6) | | 29/39 (74.4) | 21/25 (84.0) | 8/14 (57.1) |
| Cerebellar signs (%) | 45/45 (100) | 7/10 (70) | 1/7 (14.3) | 41/115 (35.7) | | 25/37 (67.6) | 16/22 (72.7) | 6/12 (50) |
| Visual abnormalities (%) | 2/38 (5.3) | 0/7(0) | 1/6(16.7) | 12/104 (11.5) | | 10/27 (37.0) | 17/23(73.9) | 2/12 (16.7) |
| Pyramidal signs (%) | 21/45 (46.7) | 7/11 (63.6) | 4/7 (57.1) | 64/128 (50) | | 31/44 (70.5) | 18/26(69.2) | 9/14 (64.3) |
| Extrapyramidal signs (%) | 10/44 (22.7) | 6/11 (54.5%) | 5/7 (71.4) | 75/127 (59.1) | | 22/42 (52.4) | 16/27 (59.3) | 4/13 (30.8) |
| PSWC (%) | 5/37 (13.5) | 4/12 (33.3) | 0/6 (0) | 14/127 (11.0) | | 38/46 (82.6) | 30/32 (93.8) | 1/14 (7.1) |
| MRI (%) | 12/41 (29.3) | 8/10 (80) | 1/7 (14.3) | 133/134 (99.3) | | 37/42 (88.1) | 28/31 (90.3) | 10/13 (76.9) |
| Location of hyperintensity | Co 8, BG 2, Th 1  (8) | Co 5, BG 3, Th 3  (5) | Co 1  (1) | Co 75, BG 22, Th 3  (75) | | Co 13, BG 14, Th 2  (16) | Co 13, BG 5, Th 2  (13) | Co 5, BG 2,  Th 1  (15) |
| Polymorphisms |  |  |  |  | |  |  |  |
| Codon 129 | 33 MM, 4 MV | 10 MM, 1 MV | 0 MM, 7 MV | 113 MM, 36 MV | | 43 MM, 0 MV | 33 MM, 0 MV | 11 MM, 2 MV |
| Codon 219 | 36 EE, 0 EK | 11 EE, 0 EK | 7 EE, 0 EK | 143 EE, 1 EK | | 41 EE, 2 EK | 32 EE, 1 EK | 12 EE, 0 EK |
| Family history |  |  |  |  | |  |  |  |
| Possible^a^ | 38 (84.4) | 10 (83.3) | 6 (75) | 9 (6.0) | | 24 (52.1) | 0 (0) | 1 (7.1) |
| Definite^b^ | 18 (40.0) | 4 (33.3) | 3 (37.5) | 1 (0.7) | | 8 (17.4) | 0 (0) | 0 (0) |

BG, basal ganglia; Co, cerebral cortex; Th, thalamus

^a^A ‘possible’ family history is one in which a member of the patient’s family had a prion disease (mutation unknown) or had dementia due to neurodegenerative disease.

^b^A ‘definite’ family history is one in which a member of the patient’s family has the same *PRNP* mutation as the patient’s
